# Beta-Amyloid Enhances Vessel Formation in Organotypic Brain Slices Connected to Microcontact Prints

**DOI:** 10.3390/biom14010003

**Published:** 2023-12-19

**Authors:** Katharina Steiner, Christian Humpel

**Affiliations:** Laboratory of Psychiatry and Experimental Alzheimer’s Research, Medical University of Innsbruck, 6020 Innsbruck, Austria; k.steiner@i-med.ac.at

**Keywords:** brain vessel, laminin, collagen microcontact print, organotypic brain slices, beta-amyloid

## Abstract

In Alzheimer’s disease, the blood–brain barrier breakdown, blood vessel damage and re-organization are early events. Deposits of the small toxic peptide beta-amyloid (Aβ) cause the formation of extracellular plaques and accumulate in vessels disrupting the blood flow but may also play a role in blood clotting. In the present study, we aim to explore the impact of Aβ on the migration of endothelial cells and subsequent vessel formation. We use organotypic brain slices of postnatal day 10 wildtype mice (C57BL/6) and connect them to small microcontact prints (µCPs) of collagen. Our data show that laminin-positive endothelial cells migrate onto collagen µCPs, but without any vessel formation after 4 weeks. When the µCPs are loaded with human Aβ40, (aggregated) human Aβ42 and mouse Aβ42 peptides, the number and migration distance of endothelial cells are significantly reduced, but with a more pronounced subsequent vessel formation. The vessel formation is verified by zonula occludens (ZO)-1 and -2 stainings and confocal microscopy. In addition, the vessel formation is accompanied by a stronger GFAP-positive astroglial formation. Finally, we show that vessels can grow towards convergence when two opposed slices are connected via microcontact-printed lanes. In conclusion, our data show that Aβ promotes vessel formation, and organotypic brain slices connected to collagen µCPs provide a potent tool to study vessel formation.

## 1. Introduction

### 1.1. Cerebrovascular Pathology in Alzheimer’s Disease

Accumulating evidence implies that overlapping cerebrovascular (e.g., arteriosclerosis, microinfarcts, intracerebral hemorrhage) and neurodegenerative pathologies drive the cognitive impairment and subsequent dementia in the ageing brain [1]. Cerebral amyloid angiopathy (CAA) is a small vessel disease and affects small arterioles and capillaries of the leptomeninges and cerebral cortex and confounds Alzheimer’s disease (AD) in an estimated 85–95% of all cases [2]. The clearest instance of a link between cerebrovascular–neurodegenerative processes is the shared beta-amyloid (Aβ) deposition in CAA and AD [3]. In AD, aggregated Aβ peptides are the primary constituent of plaques in the brain’s parenchyma. In CAA, Aβ peptides also accumulate in the walls of cerebral arterioles and capillaries, resulting in the loss of vascular smooth muscle cells and the subsequent loss of vasoreactivity [4].

### 1.2. Endothelial Cells, Angiogenesis and Aβ

Endothelial cells (ECs) form the inner layer of the vessel walls and are anchored by the basement membrane on the basal side of the endothelial tissue. Depending on the localization, ECs are enwrapped either by pericytes (along capillaries) increasing the vessel stability, or by smooth muscle cells (along arterioles, arteries) supporting the vascular tone regulation. Astrocytes cover most of the blood vessels and help to modulate blood-brain barrier (BBB) homeostasis. This neurovascular unit participates in cerebral blood flow control, regulation of the molecular exchange across the BBB, immune surveillance and angiogenesis [5]. The term angiogenesis refers to the formation of vessels from pre-existing capillary structures. Sprouting angiogenesis is commonly observed after brain injury and poses an important repair process [6]. The principal cell types mediating angiogenesis are resident ECs. They are able to recognize tissue hypoxia via the key reporter molecule hypoxia-inducible factor-1 (HIF-1) primarily driving angiogenesis [7]. The transcription factor HIF-1 promotes the high expression of a number of vascular growth factors in ECs such as the vascular endothelial growth factor (VEGF) that provides trophic support for angiogenesis [8]. These factors signal nearby vessels to detach the pericytes and degrade the local basement membrane. Then, ECs form vessel sprouts through migration towards the guiding cues and vessel lumen through proliferation in a coordinated manner. In parallel, ECs recruit pericytes and smooth muscle cells arising in a stable and functional vessel.

Aβ deposition in cerebral vessel walls is predominately composed of the more soluble Aβ40 species. However, vascular deposits also contain Aβ42, but the Aβ40:Aβ42 ratio is higher, as in plaques [9]. Opposed to this, the more toxic Aβ42 with two additional C-terminal residues preferentially accumulates in plaques. Although the key mechanism, which primarily drives the progression of AD, remains unclear, mounting evidence points to a central role of Aβ interfering with cerebrovascular pathways. The role of Aβ in angiogenesis has been discussed controversially in the literature for many years. Some reported an Aβ dose-dependent reduction in angiogenesis [10,11], while others observed angiogenesis starting near Aβ plaques [12], a denser brain capillary network [13] and even detected a molecular signaling pathway of Aβ in inducing angiogenesis [14,15,16]. It seems that AD implicates modified angiogenesis and angiogenic pathways and is considered as a potential therapeutic for AD.

### 1.3. Organotypic Brain Slices and Vessel Formation

Organotypic brain slice cultures emerged as a relatively straightforward method to study a cytoarchitecturally intact three-dimensional model of the brain ex vivo. They provide a powerful bridge combining the manipulability of in vitro models with the physiologic integrity of in vivo models. In contrast to cell cultures, organotypic brain slice cultures enable access to all cell types in the brain inclusive of ECs that form the blood vessels. Additionally, they markedly reduce the number of animal experiments, since multiple slices (depending on slice thickness and brain region of interest) can be obtained from one brain. This also allows the investigation of several variables in one system, which potentially reduces variability in experimental setups. In the membrane interface technique, brain tissue regions of interest are cultured on a semipermeable membrane interface between a humidified atmosphere and the culture medium. Meanwhile, the membrane interface technique is broadly used in neuroscience research by a large number of research groups [17,18]. Although vessels in organotypic brain slices are no longer functional due to the absence of a blood flow, they may express and release molecular factors that may influence other cells within the brain slices. Since the three-dimensional vascular structure is well-preserved in organotypic brain slices, they provide a potent tool to investigate angiogenesis in the brain [19]. 

In this current study, we aim to induce vessel formation in hippocampal organotypic brain slices of postnatal day 10 wildtype mice (C57BL/6) along microcontact-printed lanes of Aβ. Microcontact printing enables us to immobilize different Aβ peptides in a collagen hydrogel solution and to connect with brain slices. Our data provide evidence that Aβ supports angiogenesis in the mouse brain slices. 

## 2. Materials and Methods

### 2.1. Microcontact Prints

Microcontact printing enables the transfer of a pattern consisting of biomolecules to another surface. The transfer of µm size patterns is possible with this technique, which is well established in our lab [20]. The micropatterned polydimethylsiloxane (PDMS) stamp adsorbs the collagen hydrogel solution and transfers it in a corresponding pattern to the semipermeable extra membrane upon contact. PDMS stamps were made with templates from a silicon wafer called “master”. A number of stamps were generated from one master mold, which can be used indefinitely. The design of the microcontact prints was processed in Tanner L-Edit Software 15.2. Our used pattern consisted of an array of 800 µm long lines with a width of 75 µm running parallel to each other with 80 µm spaces in between. A thin layer (5 µm) of the photoresist SU-8 was spin coated (5000 rpm for 1 min) on a silicon wafer (already coated with SiO_2_). The silicon wafers then underwent a soft bake for 3 min at 90 °C on a hot plate. The photoresist SU-8 was patterned by UV lithography via a mask with the corresponding pattern using a maskless aligner (Heidelberg Instruments, Heidelberg, Germany, MLA100). The wafer was baked again at 90 °C for 3 min, developed in a bath containing the developer mr-Dev 600 for 10 min, and thoroughly rinsed with isopropanol. The surface relief of the stamp was formed by casting and curing the liquid silicone elastomer against the silicon wafer master mold. The prepolymer of PDMS (Sylgard 184 Silicone Elastomer Kit, Dow, Seneffe, Belgium) arrived in two components. The so-called elastomer curing agent was carefully blended with the elastomer base solution in a concentration of 1:10. Then, the master mold was positioned in the center of a petri dish and the blended PDMS was poured on. Arisen air bubbles were removed by means of a desiccator connected to a vacuum pump. After being left to cure overnight at 60 °C, the solid PDMS was peeled off the master mold and the stamps were cut to size with a scalpel. 

In the present study, we used VEGF (PreproTech, Cranbury, NJ, USA, 450-32), human Aβ40 (Innovagen, Lund, Sweden, SP-BA40-1), human Aβ42 (Innovagen, Lund, Sweden, SP-BA42-1), mouse Aβ42 (Abcam, Cambridge, UK, Ab120959), full length tau (R&D Systems, Minneapolis, MN, USA, SP-495) and tau P301S (Abcam, Cambridge, UK, ab246003). The aggregation of human Aβ42 was performed as already described in detail by others [21] and us [22]. Briefly, 1 mg of human Aβ42 of the protein was dissolved in 1 mL of Tris-HCl pH 9.0 and diluted 1 + 1 with PBS + 0.05% sodium dodecyl sulfate, giving a concentration of 100 µM. This was incubated overnight at 4 °C, then diluted again 1:10 with PBS and incubated for two weeks at 4 °C, yielding a final concentration of 50 µg/mL (10 µM). The bovine collagen solution type I (Sigma-Aldrich, St. Louis, MO, USA, 804592) was crosslinked with 4arm-poly(ethylene glycole) succinimidyl succinate (4arm-PEG, Sigma-Aldrich, St. Louis, MO, USA, JKA7006) while printing. All components were kept on ice to prevent premature gel formation. First, a stock solution of 12.5 mg/mL 4arm-PEG in 10 mM phosphate-buffered saline (PBS, pH 7.2) was prepared. From this solution, 25 µL were expeditiously blended with 133 µL of collagen solution and 20 µL of 100 mM PBS. The pH of the collagen hydrogel solution was set to 7.2 via adding 1.6 µL of NaOH. Additionally, 2 µL of a fluorescent antibody (Alexa Fluor 546 anti-rat, Invitrogen, Waltham, MA, USA, A11081) was added to facilitate the visualization of the print. Then, a load of 20 µL of 10 µg/mL VEGF, 1 mg/mL human Aβ40, 1 mg/mL human Aβ42, 50 µg/mL aggregated human Aβ42, 1 mg/mL mouse Aβ42, 100 µg/mL full length tau or 100 µg/mL tau P301S was added, vortexed and spinned down. The same amount of 10 mM PBS was added in place of the proteins/peptides to produce empty collagen microcontact prints as negative controls.

Immediately after collagen hydrogel preparation, 15 µL of this solution was applied onto each micropatterned PDMS stamp. A coverslip (R. Langenbrinck, Emmendingen, Germany, 01-2126/1) was placed on top to distribute the ink solution equally on the stamp and incubated for 15 min at 37 °C to allow crosslinking of the collagen hydrogel solution. After 15 min, the coverslip was removed and used to fully strike off the remaining collagen hydrogel solution. Excess solution on the borders of the pattern was removed by filter paper without touching the printing surface or left to air dry. Then, the stamp was turned upside down, and the collagen hydrogel solution was transferred to the semipermeable extra membrane (Isopore, Merck Millipore, Darmstadt, Germany, HTTP02500) by pressing-on with 18 g coins over night at 4 °C. The position of the microcontact print was marked with small dots of permanent marker to facilitate the arrangement of the brain slices.

### 2.2. Organotypic Brain Slice Cultures

Organotypic brain slice cultures represent a physiologically relevant three-dimensional ex vivo model, which is well established in our lab [18]. To consider the gender aspect, male and female mice were equally integrated in the experiment. All experiments were approved by the Austrian Ministry of Science and Research and conformed to the Austrian guidelines on animal welfare and experimentation. All work was performed according to the principles of the three Rs (replace, reduce, refine).

**Figure 1:**
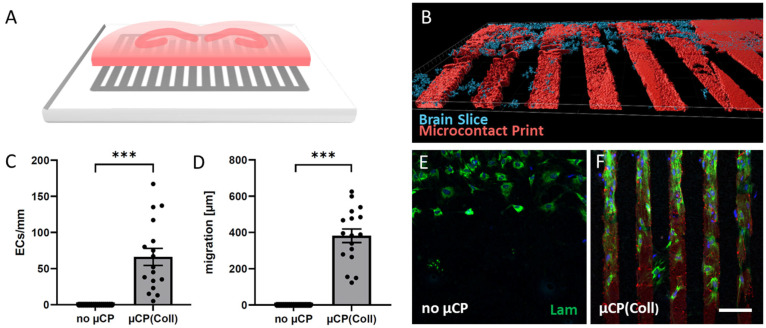
Brain slices were coupled with microcontact prints (µCPs), and the outgrowth of laminin (Lam)-positive endothelial cells (ECs) along the µCPs was measured. (**A**) A schematic representation shows a hippocampal half brain slice of a postnatal day 8-10 mouse linked to a µCP. (**B**) A confocal image of a red fluorescent µCP (Alexa Fluor 546) and a blue fluorescent (DAPI) stained brain slice was processed with the Imaris 10.0.1 software for three-dimensional representation. (**C**,**D**) The number of Lam-positive ECs per mm µCP and the distance of migration originating from the brain slice were measured on control membranes (no µCP) and collagen-only µCPs (µCP(Coll); area of analysis: 8 representative microcontact-printed lanes corresponding to an area of 600 µm in width). Values are given as mean ± SEM (*n* = 17). Statistical significance was evaluated by a two-sample *t*-test for unpaired samples (*** *p* < 0.001). (**E**,**F**) Representative microscopic images of cultures with and without µCPs. Scale bar in (**F**) = 130 µm (**E**,**F**), 80 µm (**B**).

Postnatal day 10 C57BL/6 wildtype mice were rapidly decapitated, and their brains were dissected under sterile conditions. The cerebellum was smoothly cut off and the brains were glued (Loctite 401, Henkel,Düsseldorf, Germany 231435) on their newly formed caudal surface to the sample holder platform of a water-cooled vibratome (Leica, Nussloch, Germany, VT1000S). Coronal slices at the hippocampus level with 150 μm thickness were cut in a sterile preparation medium (pH 7.2–7.3, autoclaved, 16.1 g/L MEM (Gibco, Thermo Fisher Scientific, Vienna, Austria, 11012-044), 0.43 g/L NaHCO_3_ (Merck-Millipore, Darmstadt, Germany, 144-55-8)). From each mouse, six slices were taken from the hippocampal area. These slices were horizontally cut in half and the upper hippocampal part was placed on the microcontact-printed extra membranes (Figure 1A) in cell culture inserts (Millicell, Merck Millipore, Darmstadt, Germany, PICM03050). Each well of the six-well plate (Sarstedt, Nümbrecht, Germany, 83.3920) contained 1 mL of a sterile-filtered slice culture medium (pH 7.2, sterile filtrated, 16.1 g/L MEM, 0.43 g/L NaHCO_3_, 6.25 g/L glucose (Merck-Millipore, Darmstadt, Germany, 1083371000), 116 mg/L glutamine (Merck-Millipore, Darmstadt, Germany, 1002890100), 10% heat-inactivated HS (Gibco, Thermo Fisher Scientific, Vienna, Austria, 16050-122), 25% HBSS (Gibco, Thermo Fisher Scientific, Vienna, Austria, 24020-091), 1× antibiotic-antimitotic solution (Gibco, Thermo Fisher Scientific, Vienna, Austria, 15240062)). The half slices were incubated at the microcontact prints for 4 weeks and the slice culture medium was changed once a week. In selected experiments, two opposing half brain slices with a space of 1 mm in between were cultured for 8 weeks to investigate whether vessels can grow towards one another and combine.

### 2.3. Immunostainings

Immunohistochemistry was used to evaluate the ECs and vessel formation via the basement membrane marker laminin and the tight junction protein zonula occludens (ZO)-1 and -2 and the astrocytes by the glial fibrillary acidic protein (GFAP). The cell culture inserts inclusive of the extra membrane were transferred to a six-well plate with fresh 10 mM PBS, washed 3 × 3 min and then incubated for 3 h at 4 °C in 4% paraformaldehyde (PFA, pH 7.4, filtered, 40 g/L PFA) for fixation. The extra membrane with the attached brain slice was removed from the cell culture insert and washed again for 3 × 3 min with fresh 10 mM PBS. Brain slices were incubated in Triton-PBS 0.1% (T-PBS) for 30 min at RT with soft shaking. Then, the sections were washed again for 3 × 3 min with fresh 10 mM PBS and blocked in T-PBS 0.1% + BSA 0.2% + HS 20% for 30 min while slowly shaking. With subsequent blocking, the brain slices were incubated in T-PBS 0.1% + BSA 0.2% with primary antibodies against laminin (Sigma-Aldrich, St. Louis, MO, USA, L9393, 1:500), ZO-1 (proteintech, 21773-1-AP, 1:500), ZO-2 (proteintech, 18900-1-AP, 1:100) and GFAP (Merck-Millipore, Darmstadt, Germany, AB5541, 1:2000) at 4 °C for 48 h. After washing for 3 × 3 min with fresh 10 mM PBS again, the samples were incubated with the corresponding green fluorescent Alexa Fluor 488 anti-rabbit (Invitrogen, Waltham, MA, USA, A21206, 1:400) or Alexa Fluor 546 anti-chicken (Invitrogen, Waltham, MA, USA, A11040, 1:400) in T-PBS 0.1% + BSA 0.2% at RT for 1 h while softly shaking and protected from light. Brain slices were washed for 3 × 3 min with fresh 10 mM PBS again and all slices were counterstained with 4′,6-diamidino-2-phenylindole (DAPI, Sigma-Aldrich, St. Louis, MO, USA, D9542, 1:10,000) in T-PBS 0.1% at RT for 1 h while slowly shaking and protected from light. After a last washing step of 3 × 3 min with fresh 10 mM PBS, the slices were mounted with Mowiol onto glass slides and left to dry overnight.

Widefield fluorescence microscopy was performed using the microscope Olympus (Tokyo, Japan, BX61). Alexa Fluor 488 was visualized in the green channel (ex 480/40 nm, em 527/30 nm) and Alexa Fluor 546 in the red channel (ex 535/50, em 610/75). Fluorescence images were acquired via the connected ProgRes C14 camera (Jenoptic, Jena, Germany) and Openlab Software 5.5.0. Confocal microscopy was performed using LSM 980 with Airyscan 2 (Zeiss Oberkochen, Germany). Either the 10× 0.45 NA (air) or the 25× 0.8 NA (glycerol) or the 63× 1.2 NA (glycerol) objective were selected, and the 488 nm laser was turned on to find the appropriate focus and region of interest (the border of the horizontal cutting side between the brain slice and µCP). The 63× objective was additionally corrected for differences in coverslip thickness. The imaging mode (4Y) and dyes (Alexa Fluor 488, Alexa Fluor 546, DAPI) to be detected were chosen and Airy Scan calibration was started. An interval between the first and last planes in the Z-direction was set and a Z-stack composite image was acquired in 4Y mode. For the deconvolution with the Huygens Professional software 23.10, the following parameters were used: In microscopic parameters, the sampling intervals were recalculated with the online tool microscopy Nyquist rate calculator in case they were too high. The numerical aperture of the objective was adjusted, the lens immersion was air (1) or glycerin (1.456) and the embedding medium was Mowiol (1.49). In the operations window, the signal/noise per channel was set between 10 and 20 for each channel depending on the signal quality, max. iterations to 100, variable point spread functions (PSFs) to auto and quality change threshold to 0.1%. After the deconvolution, the images were processed with the Imaris 10.0.1 software for three-dimensional representation with added surfaces.

### 2.4. Data Analysis and Statistics

A blinded image evaluation was performed to determine the number and migration distance of ECs as well as the vessel-like formation. Only ECs that clearly migrated out from the brain slice and were definitively located on the microcontact-printed stripes were considered in the quantitative analysis. Images were captured by a widefield microscope (Olympus, Tokyo, Japan, BX61 and Leica, Nussloch, Germany, DMIRB) at a 10× magnification and triple-colored to visualize green ECs with a blue nucleus on the red microcontact print. These pictures were taken from the border of the horizontal cutting side including eight representative microcontact-printed lanes (corresponding to an area of 600 µm in width). By means of ImageJ, the images were analyzed. A straight line marking the slice border was drawn, and ECs clearly outside the brain slice were counted with the multi-dot function. For determining the endothelial migration distance, the pixel-micrometer ratio scale was set according to the measurements at the microscope, and the y-coordinate of the brain slice border lane was subtracted from the y-coordinate of each multi-dot-counted EC. Values were given as mean ± standard error of the mean (SEM), absolute numbers (number of slices with vessel formation/total number of observed vessel formation) or minimum (min) and maximum (max) of the observed vessel length. The sample size (n) always specified the number of slices evaluated that were taken from different animals. Statistical significance was evaluated by a one-way ANOVA with Dunnett’s post-hoc test for comparing different treatments with the control group µCP(Col). *p*-values lower than 0.05 represented significance.

## 3. Results

### 3.1. ECs Migrate along Collagen Microcontact Prints

**Figure 2:**
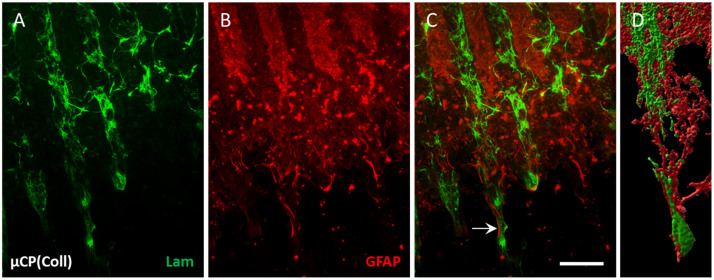
Co-localization of laminin (Lam) and glial fibrillary acidic protein (GFAP) along the collagen-only microcontact print (µCP). Hippocampal half brain slices of postnatal day 8–10 mice were coupled with collagen-only µCP (Coll) and fixed after 4 weeks in vitro. (**A**) Lam-positive endothelial cells (ECs, Alexa Fluor 488, green) migrate along the microcontact-printed lanes. (**B**) The GFAP-positive astrocytes (Alexa Fluor 546, red) organize themselves along the microcontact-printed lanes, too. (**C**) Endothelial migration (green) is accompanied by astrocytic formation (red, arrow). (**D**) A confocal zoomed-in image of the interaction between green ECs and red astrocytes along one microcontact-printed lane was processed with the Imaris 10.0.1 software for three-dimensional representation. Scale bar in (**C**) = 100 µm (**A**–**C**) 70 µm (**D**).

Organotypic half brain slices at the hippocampus level were connected to collagen-based µCPs (Figure 1A,B), and several laminin-positive ECs migrated out of the brain slice along these microcontact-printed lanes (Figure 1F). On collagen-only µCPs, on average, 66 ± 12 ECs migrated for 382 ± 37 µm (per field = eight representative microcontact-printed lanes corresponding to an area of 600 µm in width) (Figure 1C,D,F and Table 1). In contrast, slices without any µCPs showed no activation of endothelial migration at all (Figure 1C–E). The migrated ECs on collagen-only µCPs were accompanied by GFAP-positive astrocytes (Figure 2). Brain slices connected to collagen-only µCPs showed no vessel formation; however, in only 1 out of 17 brain slices, a spontaneous vessel formation (with two vessels) was seen (Table 1).

### 3.2. Effects of Alzheimer’s Proteins/Peptides on EC Activation

**Figure 3:**
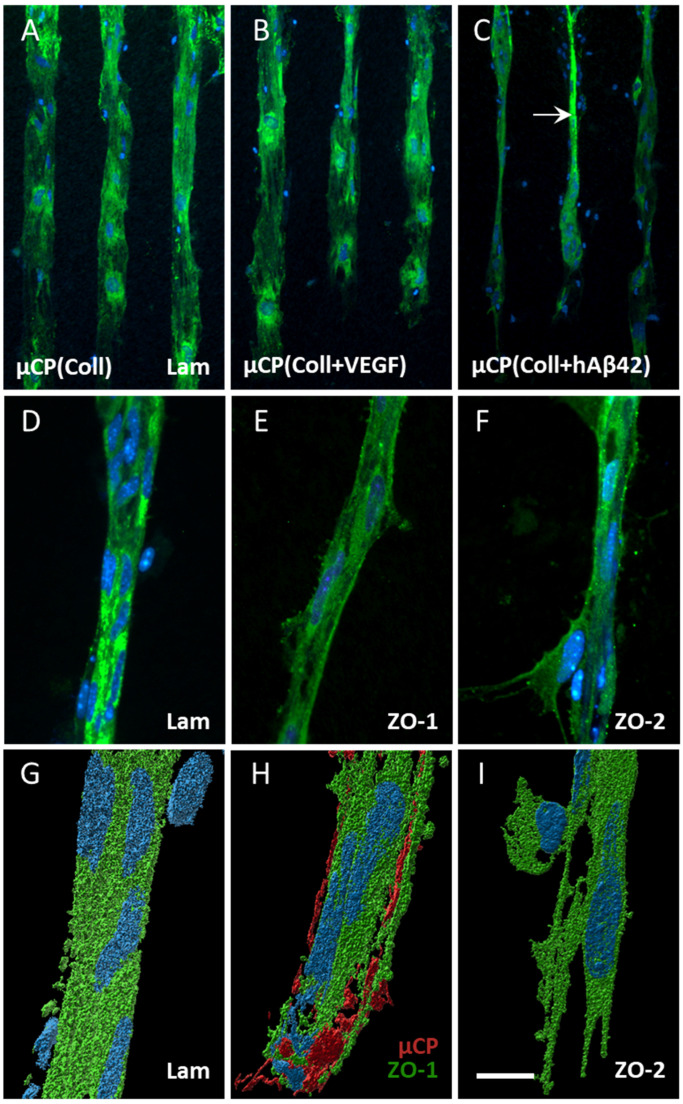
Effects of vascular endothelial growth factor (VEGF) and human beta-amyloid42 (hAβ42) on vessel formation. Hippocampal half brain slices of postnatal day 8–10 C57BL/6 wildtype mice were coupled to collagen (Coll) microcontact prints (µCPs). The slices were fixed after 4 weeks in culture and stained with laminin (Lam, Alexa Fluor 488, green), zonula occludens (ZO)-1 (Alexa Fluor 488, green), ZO-2 (Alexa Fluor 488, green) and nuclear DAPI (blue). (**A**,**B**) Endothelial cells (ECs) migrated out of the brain slice along the Coll-only µCP and along µCP loaded with VEGF but without vessel formation. (**C**) Note the vessel promoting effect of µCP(hAβ42) (arrow in (**C**)). Such vessels were positive for the basement membrane marker Lam (**D**) and the tight junction proteins ZO-1 (**E**) plus ZO-2 (**F**). (**G**–**I**) show confocal zoomed-in images of Lam-, ZO-1- and ZO-2-positive vessels, respectively, that were processed with the Imaris 10.0.1 software for three-dimensional representation. (**G**) A compact vessel with an attached cell of unknown origin. (**H**) A red fluorescent antibody (Alexa Fluor 546) was added to the solution to be printed to visualize the µCP and its interaction with an outgrown vessel. (**I**) At the vessel tip, ECs extrude their filopodia towards the printing pattern. Scale bar in (**I**) = 150 µm (**A**–**C**), 75 µm (**D**–**F**), 50 µm (**G**–**I**).

VEGF did not affect the migration of ECs, but it displayed a slight vessel formation (4 slices out of 17 showed five vessels in total; Table 1 and Figure 3B). When slices were connected to µCPs loaded with human Aβ40 or the aggregated human Aβ42, the migration of ECs significantly decreased (Table 1); however, in parallel, the vessel formation increased (4 slices out of 17 showed 6–8 vessels in total; Table 1 and Figure 3C). Mouse Aβ42 significantly decreased the migration of ECs, but no marked vessel formation was seen (Table 1). Human Aβ42, full length tau and mutated P301S tau did not affect EC migration but had a tendency to increase the vessel formation (Table 1).

### 3.3. Characterization of Vessels

Immunostainings for laminin already pointed to a vessel-like formation when stimulated with human Aβ42-loaded µCPs (Figure 3C,D). In order to characterize the vessel formation, immunostainings for ZO-1 and ZO-2 were performed and both markers clearly showed a vessel-like formation (Figure 3E,F). Confocal imaging revealed a compact laminin-positive vessel with an attached cell of unknown origin (Figure 3G). The outgrown vessel also directly interacted with the µCP, when visualizing the printing pattern via confocal imaging (Figure 3H). At the outgrown vessel tip, ECs clearly extruded their filopodia towards the printing pattern, extending the vessel formation (Figure 3I).

### 3.4. Vessel Re-Organization in Brain Slices

**Figure 4:**
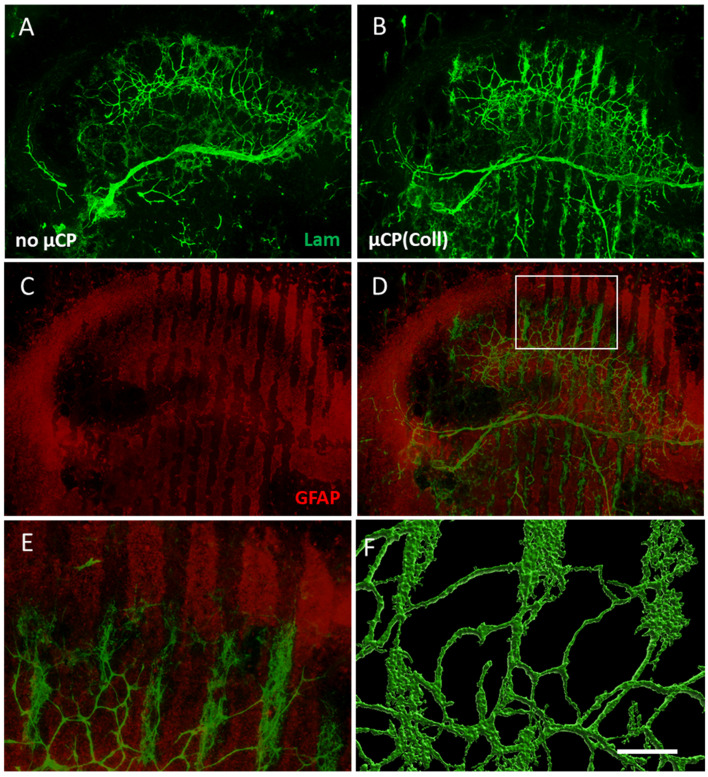
Re-organization of laminin (Lam)-positive vessels within the brain slice accompanied by astrocytic formation. (**A**) A control brain slice at the hippocampal level cultured without any microcontact print (µCP) displays a network of Lam-positive vessels (Alexa Fluor 488, green). (**B**) When such brain slices are coupled to collagen-only µCPs, the Lam-positive green vessels re-organize along these µCPs. (**C**,**D**) Likewise, glial fibrillary acidic protein (GFAP)-positive astrocytes (Alexa Fluor 546, red) re-organize and surround the Lam-positive green vessels. (**E**) A zoomed-in image of the white frame in (**D**). Note that GFAP-positive astrocytes grow along the spaces in between the microcontact-printed lanes and not on the lanes. (**F**) A confocal zoomed-in image of Lam-positive vessels was processed with the Imaris 10.0.1 software for three-dimensional representation. Scale bar in (**F**) = 550 µm (**A**–**D**), 130 µm (**E**), 80 µm (**F**).

In the control brain slices, a dense network of laminin-positive vessels was seen in the hippocampus (Figure 4A). In brain slices that were connected to collagen-only µCPs, this vessel density was markedly increased and re-organized along the collagen-only microcontact-printed lanes (Figure 4B). Co-staining with astroglial marker GFAP showed that the laminin-positive vessels were surrounded by astrocytes. Interestingly, these GFAP-positive astrocytes were not located directly on the microcontact-printed lanes but in the spaces in between, clearly supporting the growth and re-organization of vessels (Figure 4C–E). A confocal zoomed-in image revealed a re-organized vessel network that was denser along the µCPs (Figure 4F).

### 3.5. Vessel Re-Growth between Two Slices

**Figure 5:**
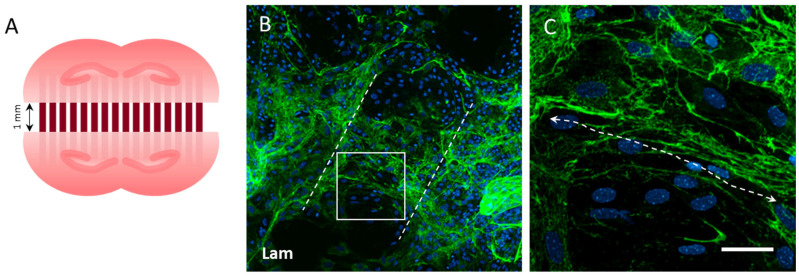
Vessel grows towards two opposing hippocampal half brain slices with a space of 1 mm in between when cultured for 8 weeks. (**A**) A schematic representation of the experimental setup: two opposing hippocampal half brain slices were connected to each other by a microcontact print (µCP) loaded with human aggregated Aβ42. (**B**) Immunostainings for laminin (Lam, Alexa Fluor 488, green) show several vessels in the brain slices (dashed white lines mark the two horizontal cuts of the half brain slices). Vessels from both brain slices grew together in a dense network, but were unaffected by the microcontact-printed lanes (**B**, co-stained with blue fluorescent nuclear DAPI). (**C**) A zoomed-in image section of the space in between (frame in **B**) shows Lam-positive network connecting both brain slices. Scale bar in (**C**) = 500 µm (**B**), 60 µm (**C**).

To investigate whether vessels from two adjacent inversely opposed half brain slices (Figure 5A) can grow together, this collocation of slices was cultured on human aggregated Aβ-loaded µCPs with a space of 1 mm. After 8 weeks in the culture, a strong laminin-positive network was found between the two brain slices (Figure 5B). A zoomed-in view verified that the two brain slices were connected, but laminin-positive vessels grew rather undirected and were not organized along the collagen µCPs (Figure 5C).

## 4. Discussion

In the present study, we showed that ECs migrated on collagen-based µCPs connected to organotypic mouse brain slices. When slices were cultured on Aβ-loaded µCPs, the number of ECs was significantly reduced with a more pronounced vessel formation.

### 4.1. Angiogenesis in Organotypic Brain Slices

Angiogenesis assays can be categorized into three main categories: (i) in vitro, (ii) ex vivo and (iii) in vivo [23]. However, none of them perfectly mimic a functional human vascular microenvironment, but rather they enable us to study specific aspects of angiogenesis. In vitro EC migration and proliferation assays may prove efficient and insightful, yet unable to consider all physiological forces. On the contrary, in vivo assays provide the most reliable information on the process of angiogenesis in an intact organism, but they are ethically questionable, expensive and require considerable technical skills. Ex vivo assays constitute a kind of compromise between in vitro and in vivo assays, combining the manipulability of in vitro models with the physiologic integrity of in vivo models.

One of the most popular ex vivo methods for studying angiogenesis to date is the three-dimensional aortic ring assay. Such sections of rodents are immersed in gels comprised of Matrigel, fibrin or collagen and subsequently cultured in media permitting vessel sprouting [24]. Another highly used ex vivo angiogenesis model is the retinal explant assay, in which isolated retina fragments from mice are embedded in a three-dimensional extracellular matrix substitute. However, to study angiogenesis in the brain, ex vivo assays are limited. The three-dimensional organotypic brain slice cultures enable access to all cell types in the brain inclusive of ECs that form the blood vessels [19].

Our lab provided evidence that ECs of capillaries survive in organotypic brain slices, and the cellular environment in the brain slice culture even enables them to reform capillaries [19]. Recently, we showed that collagen hydrogels loaded with fibroblast growth factor-2 enhanced vessel re-growth between two organotypic brain slices [25]. In the present study, we aimed to extend our previous findings and investigated whether ECs migrate in a more directed manner along a novel µCP assay. Indeed, the migration of ECs originating in the brain slice along collagen-based µCPs was sufficient to subsequently form vessels from the brain slice towards the printed lanes. Hence, organotypic brain slices connected to collagen-based µCPs seem to be a good model to study vessel formation.

### 4.2. Effects of Collagen on Vessel Formation

The extracellular matrix is of high importance for angiogenesis since it contains cues for active cellular signaling. Extracellular matrix substitutes such as Matrigel, fibrin or collagen became very popular in three-dimensional angiogenesis assays as they enable the capillary-like formation of ECs. Despite its versatility and affordability, the usage of Matrigel, a basement membrane-like matrix of tumor origin, is complex. A proteomic analysis showed that it contains more than 1800 unique proteins with unknown functions and in variable amounts, which may cause variability among experiments [26,27]. Furthermore, Matrigel cultures require growth factor supplements due to the limited ability of the aortic rings to sprout spontaneously in this dense matrix [24]. When working with fibrin gels, prepared from fibrinogen and thrombin, very fast handling is needed due to the rapid polymerization mechanism [28]. In addition to this, the culture medium of fibrin gel cultures should contain a plasmin inhibitor to suppress fibrinolysis by the aortic rings, which would rapidly destroy the matrix needed for ECs to sprout [24].

In our lab, we have extensive experience in using collagen as an extracellular matrix [29]. Collagen provides a biocompatible and non-toxic possibility to mimic both the structural and biological properties [29]. Collagen is not only responsible for the mechanical resilience of the connective tissue, but also provides strong support for cell migration, cell attachment, cell differentiation, cell proliferation and cell survival. It is broadly accepted that collagen matrices are useful for EC cell proliferation and migration, as well as for delivering pro- or antiangiogenic factors [30,31]. ECs are known to penetrate and extend sprouts into the collagen matrix [30,31], and various polymerized three-dimensional collagen matrices are frequently used to study angiogenesis by embedding or growing ECs on top [32,33]. In the present study, collagen was chemically crosslinked with PEG to assemble collagen into fibrils resulting in gel formation. Such collagen hydrogels were stable in the culture for up to 9 days, but stability markedly decreased after 14 days in the culture, gradually releasing the loaded molecules [34]. Our results point out that collagen alone provided a good basement membrane-like substrate sufficient to stimulate EC migration from the brain slice towards the printed lanes, but not sufficient to induce vessel formation.

### 4.3. Effects of Aβ on Vessel Formation

Growing evidence suggests that perturbed angiogenesis plays a critical role in the pathogenesis of AD. It is well known that Aβ40 is processed and released from blood platelets, possibly implicated in blood clot formation preventing excessive bleeding when a blood vessel is injured, but also in CAA [35,36]. Further, the more toxic Aβ42 may not only be involved in plaque progression, but also in vessel re-organization [35]. However, the role of these Aβ peptides is discussed controversially in the process of angiogenesis. Some could not find an effect of Aβ on the vascular density at all [15], while others, on the contrary, showed that angiogenesis started near Aβ plaques [12]. Some groups observed an Aβ dose-dependent reduction in angiogenesis and higher vessel disruption in the presence of Aβ [10,11], whereas others reported that Aβ40 and Aβ42 stimulated the formation of capillary-like structures [13,37]. In the present study, we show that human Aβ40 or the aggregated human Aβ42 loaded to the µCPs significantly decreased the migration of ECs; however, in parallel, the vessel formation increased. Non-aggregated human Aβ42 µCPs showed a similar tendency (decreased migration plus enhanced vessel formation), though not reaching statistical significance. We verified the proper aggregation of human Aβ42 in a previous study [22] using an adapted method from Ryan and colleagues [21]: human Aβ42 appears at 4 kDa in the Western blot, whereas aggregated human Aβ42 shows a smear of higher 40–80 kDa aggregated species [22]. Our data suggest that human Aβ42 monomers had less strength to reduce EC migration and to form vessels compared to Aβ42 aggregates. In contrast, mouse Aβ42 monomers had even stronger effects on reducing EC migration compared to human Aβ42. Using mouse Aβ42 monomers, on average only 10 ECs per field were found, probably because the number of ECs to induce sufficient vessel formation was too small. Another reason for the observed differences between human and mouse Aβ42 might be the difference between murine and human Aβ in three amino acids at the residues 5, 10 and 13 [38]. Due to this fact, murine Aβ tends to have lower aggregation in comparison to human Aβ. We have no indication that collagen affects the Aβ structure, but we cannot exclude the possibility that the soluble Aβ monomer also aggregates in the collagen hydrogel over time, which must be explored by crystallography or electron microscopy [22]. Our data clearly point to a vessel-forming activity of Aβ; however, the exact mechanism cannot be explained. It seems possible that Notch signaling may play a role, as altered Notch signaling due to higher Aβ levels has been reported to increase endothelial sprouting [14,16,39]. Definitively, more work is necessary to evaluate how Aβ can activate ECs and subsequent vessel formation, especially in AD. In the past decades, several transgenic mouse models of CAA have been developed [40]; however, to our knowledge, there exists no in vitro model of CAA so far. Thus, combining organotypic brain slices with Aβ-loaded µCPs may represent a novel simple in vitro CAA model.

Since, beyond Aβ plaques, tau pathology is also a driving factor of AD, we aimed to compare the Aβ effects with tau-loaded µCPs. Tau is a microtubule-associated protein that is predominantly found in the cytosol and axons of neurons. AD is characterized by intracellular neurofibrillary tangles (NFTs) composed of hyperphosphorylated tau protein. Although noticeably less discussed, tau oligomers were also found to accumulate in the cerebral microvasculature of human patients with AD [41]. In tau transgenic mice with striking NFT pathology, NFTs promoted increased numbers of small-diameter blood vessels that appeared to have tight turns and spiral paths in addition to the increased expression of angiogenesis-related genes in endothelial cells [42]. Based on these results, we loaded full length tau as well as mutated P301S tau to the µCPs. Both full length tau and mutated P301S tau did not significantly affect EC migration, but clearly had a tendency to increase the vessel formation. However, the mechanism whereby tau induces these changes is unclear.

Finally, as a positive control, we wanted to explore the role of the well-known angiogenetic growth factor VEGF [18]. Unfortunately, the addition of VEGF to the collagen-based µCPs did not increase the number and distance of migrated ECs compared to collagen-only µCPs. However, when VEGF was loaded to the µCPs, there was a minimal increase in vessel formations, comparable to the Aβ-loaded µCPs. VEGF is known for its important pro-angiogenic activity, also in organotypic brain slice cultures [18]. Again, the interplay between varying concentration levels of VEGF and subsequent Notch signaling is important for EC differentiation in physiological angiogenesis [43].

### 4.4. Characterization of Vessel Formation

Laminin is a non-collagenous connective tissue protein that is the major functional constituent of basement membranes underlying the endothelial cell layer. It is a well-established marker for staining blood vessel walls [44] and has been successfully used in organotypic brain slices [19,25,45]. In fact, we were able to show laminin-positive ECs due to their endogenous laminin production as well as compact laminin-positive vessels along the µCPs beyond the brain slice. To further support vessel formation, we used antibodies against ZO-1 and ZO-2, two tight junction proteins. ZO-1 and ZO-2 are peripheral membrane proteins that bind to the transmembrane proteins occludin and claudin, linking them to the cytoskeletal actin. Actually, ZO-1 may be involved in regulating EC proliferation and differentiation [46] and has been successfully used as an EC marker in organotypic brain slices [47]. ZO-1 as well as ZO-2 show a pronounced membrane-associated peripheral staining and are clearly apparent along our vessel formations.

We reported vessel formation along µCPs (i) brain slice external, (ii) brain slice internal and (iii) between two brain slices. These results indicate that collagen-based µCPs have the power to remodel the vascular network of organotypic brain slices. At the tips of the newly formed vessels, ECs clearly extruded their filopodia towards the µCPs: the guiding cues. When we visualized the µCPs, vessel formation occurred indeed directly on the µCP lanes. Furthermore, we detected attached cells of unknown origin along the newly formed vessels. They may be either pericytes or smooth muscle cells recruited by ECs. In any case, this further implicates an angiogenetic process. We further showed that astrocytes accompanied the EC migration that originated in the brain slice. This also gives an indication of effective angiogenesis, since astrocytes are known to cover most of the blood vessels and modulate BBB homeostasis, supporting the migration and providing more stability to the newly formed vessels. Taken together, we are confident that angiogenetic processes occur along the µCPs. It remains unclear if the vessels are functionally active.

### 4.5. Limitations and Translation to Humans

This study provides a proof-of-concept that collagen-based µCPs can be used as an extracellular matrix for the proliferation and migration of vascular ECs originating from organotypic brain slice cultures. This study also shows some limitations, though. (i) We have already shown in previous studies that collagen continuously degrades at variable rates in the culture. Such inconsistent collagen degradation processes may have caused variability in our experimental setup, which may explain in part the statistically non-significant vessel formation. (ii) We observed strong EC migration originating in the brain slice along collagen-based µCPs, but a subsequent vessel formation was a rather rare event. The lack of generating a gradient of the biomolecules loaded to the µCPs may also play a role, since varying VEGF levels in synergy with other growth factors tightly regulate EC migration as well as vessel formation [43]. As collagen-based µCPs degrade over time, they do gradually release the loaded biomolecules, but probably in a less controlled manner. (iii) Previous results from our lab revealed that there is a time-dependent increase in laminin-positive cells in the location of the collagen hydrogel from 2 to 8 weeks [25]. When collagen hydrogel was microcontact-printed, we were not able to observe migrated ECs along the µCPs after 8 weeks, but after 4 weeks. Interestingly, EC sprouts of aortic rings embedded in collagen form already after 3 days and tend to regress after 10 days in the culture [30,31]. However, organotypic brain slices need at least 2 weeks in the culture to flatten down and to become transparent, increasing the quality of immunostaining and microscopic analysis. Whether a shorter culture period of only 2 weeks would provide benefits needs to be assessed. (iv) As vessels may need a steady applied support, it is also possible that the accompanied astroglial re-formation could enhance and stabilize vessel formation. To increase the number of vessel formation, we will incorporate glial factors into the collagen hydrogel solution in future studies. (v) The majority of neurodegenerative diseases like AD develop in ageing adult individuals. However, brain slices for cultures are commonly taken from postnatal day 8–10, since the cytoarchitecture is already established at this age and cellular survival is higher. It would be of interest to study Aβ effects in adult wildtype brain slices in long-term studies, though challenging due to the extensive cell loss apparent in adult brain slices. The age of onset in most transgenic CAA models is rather long [40], which poses again the challenge of culturing adult brain tissue. For this reason, we induced CAA in adult wildtype mice via transcardial infusion of labeled Aβ, and subsequently cultured adult brain slices in vitro [48]. Indeed, Aβ was found in some vessels representing an in vitro CAA model. Our present brain slice model of Aβ-loaded µCPs may represent a simple CAA model but needs further characterization.

Our data provide evidence that the Aβ pathology in AD enhances vessel formation to possibly compensate Aβ plaque-driven damages. In the APP transgenic mouse model of AD, it has been shown that increased angiogenesis ameliorates memory impairment [49]. We were able to induce vessel re-growth between two slices via Aβ-loaded µCPs, though vessel functionality remains unclear. Nevertheless, collagen-based µCPs may provide an effective tool in brain vessel repair, serving as a structural support and providing a permissive microenvironment. To demonstrate the potential of collagen-based µCPs in brain vessel repair, more in vivo experiments are necessary. The Aβ plaque pathology most probably disrupts small vessels in the brain, and there are mechanisms to counteract this process. In fact, it seems likely that an analogous situation also occurs in human AD brains. Indeed, denser and longer capillary networks with preserved pericytes were found in human AD cohorts when compared to the non-demented controls [50,51]. It is suggested that changes in the human capillary network may be related to the atrophy in the affected regions and may compensate for the cognitive impairment to a certain degree.

## 5. Conclusions

Here, we report that our brain slice model connected to µCPs is attractive to elucidate the cellular and molecular mechanisms of angiogenesis, to identify pro- or antiangiogenic agents or to screen for potentially effective drugs in angiogenesis-dependent diseases. In conclusion, our data show that ECs migrate on collagen-based µCPs when connected to organotypic mouse brain slices from mice. When slices were cultured on Aβ-loaded µCPs, the number of ECs was reduced, but a higher vessel formation was observed. Our data provide evidence that specific forms of Aβ can induce vessel formation and may be implicated in the pathophysiology of AD.

## Figures and Tables

**Table 1:** Quantitative analysis of slice-derived endothelial cell migration and vessel formation along collagen-loaded microcontact prints (µCPs).

Treatment	n	ECs per mm	ECs Migration [µm]	Slices with New Vessels/Total Vessel Count	Vessel Length [min–max] in µm
µCP(Col)	17	66 ± 12 vs.	382 ± 37 vs.	1/2 (12%)	411
µCP(Col + VEGF)	17	66 ± 15	292 ± 25	4/5 (29%)	104–476
µCP(Coll + hAβ_40_)	17	21 ± 7 *	244 ± 38 *	4/6 (35%)	144–310
µCP(Col + hAβ_42_)	17	55 ± 15	359 ± 36	4/5 (29%)	172–509
µCP(Col + hAβ_42 agg_)	17	24 ± 5 *	210 ± 25 **	4/8 (47%)	118–607
µCP(Col + mAβ_42_)	17	10 ± 3 **	188 ± 15 **	1/1 (6%)	140
µCP(Col + tau _fl_)	20	39 ± 11	273 ± 27	5/8 (40%)	111–678
µCP(Col + tau _P301S_)	17	54 ± 18	300 ± 39	3/7 (41%)	147–269

Hippocampal vibrosections (150 µm, half slices) of postnatal day 8–10 C57BL/6 wildtype mice were cultured for 4 weeks on microcontact prints (µCPs) loaded with vascular endothelial growth factor (VEGF), beta-amyloid (Aβ), tau and without any load as control. Sections were then fixed and immunohistochemically stained for laminin and zonula occludens-1/2 using Alexa Fluor 488. The number of endothelial cells (ECs) per mm microcontact print, the migration distance from the brain slice, the number and length of outgrown vessels were measured along 8 representative microcontact-printed lanes (corresponding to an area of 600 µm in width). Values are given as mean ± standard error of the mean (SEM), absolute numbers (number of slices with vessel formation/total number of observed vessel formation), minimum (min) and maximum (max) of observed vessel length. Statistical significance was evaluated by one-way ANOVA with Dunnett’s post-hoc test for comparing different treatments with the control group µCP(Col) (vs. = versus, * *p* < 0.05; ** *p* < 0.01).

## Data Availability

The data that support the findings of this study are available upon request from the corresponding author.
